# A case of TEVAR for acute aortic dissection after MICS AVR and retroperitoneal tumor resection

**DOI:** 10.1093/jscr/rjab559

**Published:** 2021-12-23

**Authors:** Masahiro Tsutsui, Masahiko Narita, Ryohei Ushioda, Yuta Kikuchi, Tomonori Shirasaka, Natsuya Ishikawa, Hiroyuki Kamiya

**Affiliations:** Department of Cardiac Surgery, Asahikawa Medical University, Asahikawa, Hokkaido, Japan; Department of Cardiac Surgery, Asahikawa Medical University, Asahikawa, Hokkaido, Japan; Department of Cardiac Surgery, Asahikawa Medical University, Asahikawa, Hokkaido, Japan; Department of Cardiac Surgery, Asahikawa Medical University, Asahikawa, Hokkaido, Japan; Department of Cardiac Surgery, Asahikawa Medical University, Asahikawa, Hokkaido, Japan; Department of Cardiac Surgery, Asahikawa Medical University, Asahikawa, Hokkaido, Japan; Department of Cardiac Surgery, Asahikawa Medical University, Asahikawa, Hokkaido, Japan

**Keywords:** acute aortic dissection, iatrogenic, TEVER

## Abstract

If multiple treatments are performed within a short time, when something occurs, it is difficult to identify its cause. Here, we present a case of thoracic endovascular aortic repair (TEVAR) for acute aortic dissection (AAD) after multiple treatments. A 76-year-old woman underwent minimally invasive aortic valve replacement, transcatheter lumbar artery embolism and retroperitoneal tumor resection within a short period of time. After a series of procedures, the patient experienced sudden back pain, and computed tomography revealed an AAD Type B. Her back pain persisted; therefore, we performed TEVAR, and the post-operative course was uneventful. In this case, the relationship between AAD and treatment before AAD was unclear, but AAD should considered when performing treatments that may cause AAD.

## INTRODUCTION

Multiple treatments within a short period of time are needed if various diseases occur simultaneously. When a new disease occurs after short-term treatment, it is difficult to identify the cause of the new disease, including iatrogenic disease. Here, we present a case of post-multiple-treatment acute aortic dissection (AAD).

## CASE REPORT

**Figure 1:**
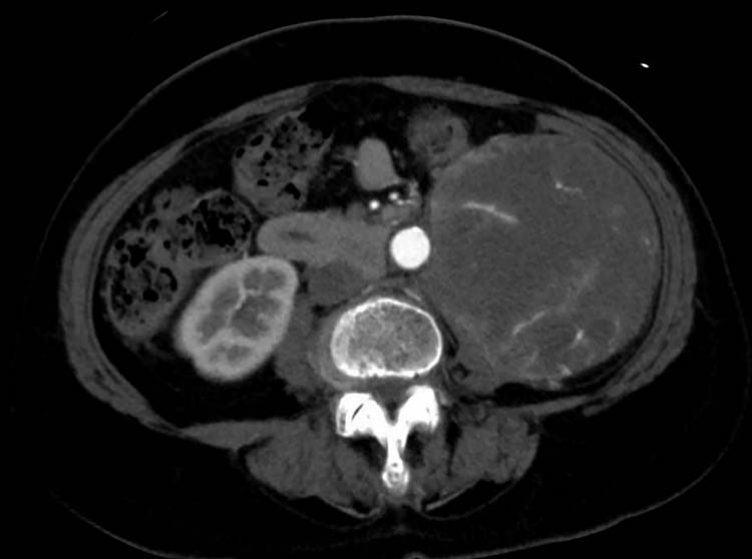
A giant retroperitoneal tumor.

**Figure 2:**
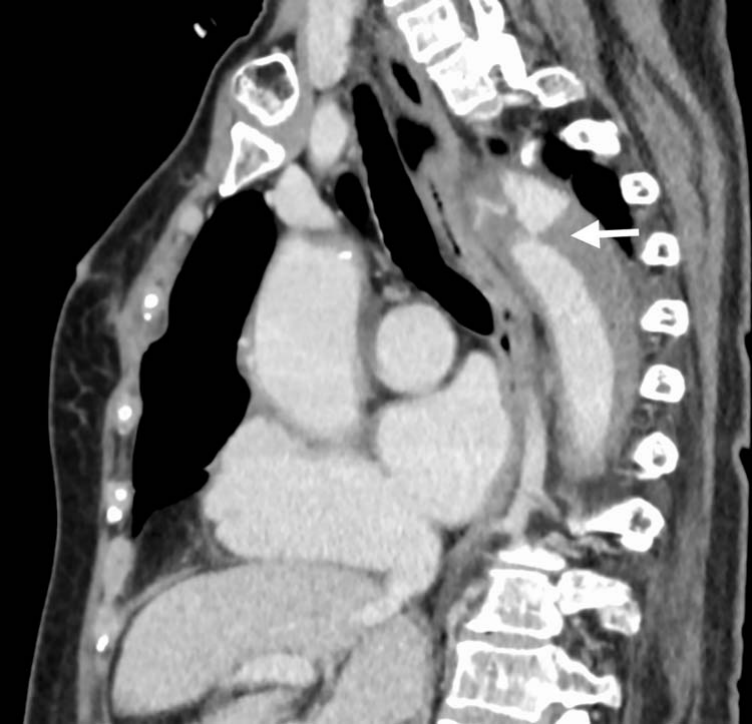
Arrow shows ULP at the distal arch.

**Figure 3:**
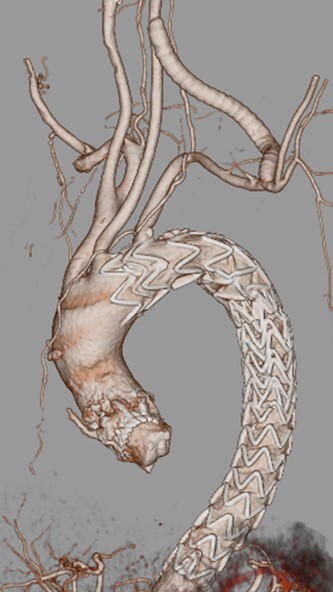
After TEVAR 3DCT VR.

**Figure 4:**
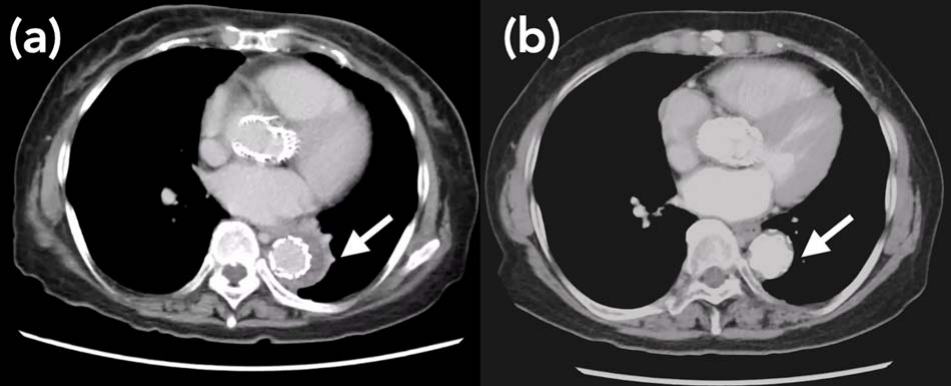
Arrow shows false lumen. (**a**) One week after TEVAR. False lumen still remains. (**b**) One year after TEVAR. False lumen has almost disappeared.

A 76-year-old woman with a giant retroperitoneal tumor was scheduled for surgical resection (Fig. 1). However, preoperative examination revealed severe aortic valve stenosis (AS); therefore, the patient first underwent minimally invasive aortic valve replacement (MICS-AVR) for severe AS. In this operation, we approached the surgical site through the right third intercostal space, and cardiopulmonary bypass was established with right femoral arterial and venous cannulation. PERCEVAL (Livanova, London, UK), a sutureless valve, was used as the artificial valve. Five days post-operatively, she complained of back pain, and a computed tomography (CT) scan revealed bleeding in the tumor and tumor growth. There were no findings of aortic dissection. A peripheral branch of the left third lumbar artery seemed to be the bleeding site; thus, lumbar artery transcatheter artery embolism (TAE) was performed. Although the bleeding stopped, early surgery was considered to be appropriate for the tumor. Therefore, retroperitoneal tumor resection and left nephrectomy were performed on the 12th post-operative day. Four days after tumor resection, the patient suddenly experienced persistent back pain again. CT revealed a Type B AAD. The aorta was dissected from the distal part of the left subclavian artery (LSA) to the level of the right renal artery, and an ulcer-like projection (ULP) was observed at the distal arch (Fig. 2). Although conservative treatment was started immediately, her back pain persisted and follow-up CT revealed slight growth of the ULP. Thus, we decided to perform thoracic endovascular aortic repair (TEVAR) using Valiant (Medtronic, Minneapolis, USA). Before the TEVAR procedure, we performed extra-anatomical bypass from the left common carotid artery to the LSA using PROPATEN (Gore, USA). The Valiant was deployed from Zone2 to just above the celiac artery (Fig. 3). The post-operative course was uneventful, and the patient was discharged without any complications. One year after discharge, CT showed that the aorta was remodeled almost as before (Fig. 4).

## DISCUSSION

We experienced a Type B AAD in a patient who had received several treatments. In this case, MICS-AVR with right femoral arterial cannulation, TEA and subsequent resection of the retroperitoneal tumor were performed within a short time, and each procedure seemed to have the potential to causing iatrogenic acute aortic dissection (IADD). IADD due to cardiovascular treatment or examination has an incidence rate of 0.06-5.8% [1–4]. Cardiovascular surgery is one of the causes of IADDs. Ram *et al*. reported that femoral artery cannulation during cardiac surgery was a significant risk factor of IADD [1]. Williams *et al*. had the same opinion after analyzing the STS data [2].

When the dissection is attributed to femoral arterial cannulation, it may be related to direct trauma by the cannula or because of the jet effect of the cannula lifting an atherosclerotic plaque in the aorta [1]. In our case, it is unlikely that it was a direct injury, but we cannot deny that the jet effect caused dissection.

Although there are no reports of IAAD due to TAE for the lumbar artery, endovascular operation using a catheter, in particular coronary angiography and transcatheter valve replacement, is one of the causes of IADD [1]. Sasaki *et al*. reported a case of abdominal aortic dissection during transfemoral angiography due to left renal hematuria [5]. Catheter operation of the abdominal aorta seems to also be capable of causing aortic dissection in rare cases.

Angeles *et al*. reported a rare case of aortic dissection after para-aortic lymphadenectomy. They concluded that aortic dissection should be considered as a potential complication after para-aortic lymphadenectomy [6]. In our case, retroperitoneal tumor resection and left nephrectomy were performed, and the surgical site was close to the aorta; however, para-aortic lymphadenectomy was not performed.

In summary, in this case, the relationship between AAD and the three different procedures was unclear; therefore, the potential of IAAD seemed to be low.

However, aortic dissection should be considered when performing treatments that may cause IAAD. Moreover, if IAAD occurs, the selection and timing of treatment should be carefully considered.
